# (Un)Equitable distribution of health resources and the judicialization of healthcare: 10 years of experience in Brazil

**DOI:** 10.1186/s12939-019-0914-5

**Published:** 2019-06-03

**Authors:** Luciana de Melo Nunes Lopes, Francisco de Assis Acurcio, Semíramis Domingues Diniz, Tiago Lopes Coelho, Eli Iola Gurgel Andrade

**Affiliations:** 1 Faculty of Medicine, Federal University of Minas Gerais, 190 Professor Alfredo Balena Avenue, Santa Efigênia, Belo Horizonte, Minas Gerais 30130-100 Brazil; 2 Faculty of Pharmacy, Federal University of Minas Gerais, 6627 Presidente Antônio Carlos Avenue, São Luiz, Belo Horizonte, Minas Gerais 31270-901 Brazil

**Keywords:** Judicialization of healthcare, Public health, Health policy, Health equity

## Abstract

**Background:**

Equity has been acknowledged as a required principle for the fulfilment of the universal right to health once it seeks to tackle avoidable and unfair inequalities among individuals. In Brazil, a country marked by iniquities, this principle was adopted in the Brazilian National Health System (SUS) organization. But the phenomenon known as judicialization of healthcare, anchored in the argument of universality of the right, has been consolidated as a health policy parallel to the SUS. The analysis of these lawsuits distribution according to their beneficiaries’ socio-economic profile can contribute to the verification of the judicialization’s potential for reducing inequalities, thus becoming an auxiliary activity in the fulfilment of the universal and egalitarian right to health. This study aimed to assess what socioeconomic factors are associated to municipalities that had larger numbers of beneficiaries from lawsuits in health in the state of Minas Gerais, Brazil, from 1999 to 2009.

**Methods:**

It is a descriptive quantitative study of the residence municipalities of beneficiaries registered in database regarding all deferred lawsuits against the state of Minas Gerais from 1999 to 2009. The verification of cities’ socio-economic profile was performed based on information of the Brazilian Institute of Geography and Statistics’ 2010 Demographic Census and on indexes derived from it. The variables studied for each municipality were: number of beneficiaries; resident population; Social Vulnerability Index (IVS); and Municipal Human Development Index (IDHm). Descriptive and statistical analysis were used to verify factors associated with a larger number of beneficiaries in a municipality.

**Results:**

Out of 853 municipalities in Minas Gerais, 399 were registered as residence of at least one of the 6.906 beneficiaries of studied lawsuits. The residence non-information index was 11,5%. The minimum number of identified beneficiaries living in a municipality was 1 (one) while the maximum was 1920. The binary logistic regression revealed that high and very high IDHm (OR = 3045; IC = 1773-5228), IVS below 0.323 (OR = 2044; IC = 1099- 3800) and population size above 14.661 inhabitants (OR = 6162; IC = 3733-10,171) are statistically associated to a greater number of beneficiaries of lawsuits in health within a municipality.

**Conclusions:**

The judicialization of health care in Minas Gerais, from 1999 to 2009, didn’t reach the most vulnerable municipalities. On the contrary, it favored a concentration of health resources in municipalities with better socioeconomic profiles. The register of all beneficiaries’ municipalities of residence as well as individual socioeconomic data can contribute to a more conclusive analysis. Nevertheless, in general, the results of this study suggest that the judicial health policy conducted from 1999 to 2009 was not an auxiliary tool for the fulfilment of an equitable right to health in Minas Gerais.

## Background

The fundamental right to health was established in Brazil by the Federal Constitution of 1988, which declared health as a universal right and a State duty [1]. To ensure the right to health, the Brazilian Constitution created the Brazilian National Health System (SUS), based on the principles of universality, comprehensiveness and equity [1, 2].

SUS’ legal framework expressly recognizes the social determination of the health-disease process, which points to the importance of assuming our social organization structure as a decisive aspect for the fulfillment of the right to health [3]. Appreciating concrete aspects of Brazil’s reality, Victora [4] points that the creation of SUS is considered one of the main causes of health status evolution of the Brazilian population [4, 5]. From 1990 to 2007, child mortality rate declined 58% and life expectancy rose from 66,6 years in 1990 to 72,8 years in 2008 [5].

However, parallel to the Brazilian public health system development process, citizens began to seek the assurance of the constitutional right to health, especially regarding the access to medicines, via the Judiciary [2, 6]. This phenomenon of suing SUS to request free access to health services and goods has been called “the judicialization of healthcare”. It has exponentially grown over the last two decades, becoming object of attention of several social actors [7]. Although Brazil is the most notorious country in studies and publications regarding the judicialization of healthcare [8], it has also been intensified in other places [8, 9]. In Latin America, the Judiciary has increasingly assumed the role of interpreting and protecting the human rights and has even obliged governments to redefine health policy priorities. Within the region, individual lawsuits are the large majority and judicial decisions are usually favorable to health claims without further investigation about their impact on the health policy as a whole [8].

The expenditure with judicial health demands in Brazil have grown and significantly impacted on the organization of SUS [10–13]. From 2008 to 2015, the Federal public expenditure on complying with judicial health decisions rose 1006% [13]. These unscheduled expenditures generate administrative challenges that, according to experts, potentially enlarge access to healthcare inequities [8, 11, 12] due the redirection of health resources regardless of the priorities of public health [12].

Assuming that 1) health resources distribution is decisive for establishing an equitable policy [14] and 2) the judicialization of healthcare interferes in the redistribution of health resources [10, 11, 13, 15], it becomes essential to investigate if the set of judicial decisions on health has favored a concentration or a deconcentration of health resources. Have places with better socioeconomic conditions been benefited from the judicialization of healthcare? This study aims to assess what socioeconomic factors are associated to municipalities that had larger numbers of beneficiaries from lawsuits in health in the state of Minas Gerais, Brazil, from 1999 to 2009.

## Methods

This is a quantitative descriptive study based on registers of the 6.112 deferred lawsuits sued against the Health Secretary of the State of Minas Gerais, Brazil, in the period of October of 1999 to October of 2009. The database was built by the Federal University of Minas Gerais’ Research Group in Health Economics (GPES/UFMG) from the information provided by the state of Minas Gerais. The variables registered in the database are about the lawsuit (number, date, court, kind of lawsuit, etc.), the beneficiary (municipality of residence, gender, profession, age, etc.), the author (if public defense, prosecution service, etc.), the judicial representative (kind, professional register, etc.), the defendant (government sphere), the medical care (information of health professionals, prescriptions, diagnostics, etc.), the drug (name, concentration, dosage, insertion in SUS’ official list, etc.) and about the procedures and materials (name, quantity, etc.). This database has been updated but, due to the extensive number of lawsuits to be explored, robust information after 2009 is not available yet. To conduct this study, all beneficiaries’ municipalities of residence were considered.

The verification of the municipalities’ socioeconomic conditions was based on information of the Brazilian Institute of Geography and Statistics’ (IBGE) 2010 Demographic Census and on two indexes derived from it that were defined and disclosed by the Institute of Research in Applied Economics (IPEA) of Brazil.

The dependent variable analyzed for each municipality was the number of beneficiaries of lawsuits in health from 1999 to 2009 while the independent variables were: the resident population in 2010, the Social Vulnerability Index (IVS) 2010 and the Municipal Human Development Index (IDHm) 2010. Detailed information about the two indexes disclosed by IPEA are provided below:

The IDHm aims to adapt the global IDH methodology to Brazilian municipalities. It is composed by the same three components of IDH: longevity (measured by life expectancy at birth), education (measured by adult population schooling and young population school flow) and income (measured by per capita income). The IDHm, which ranges from 0 to 1, enables the comparison of Brazilian municipalities over time and facilitates the orientation of interventions to improve municipalities’ socioeconomic conditions. The range of municipal human development measured by the index is: very low (0–0,499), low (0,500-0,599), medium (0,600-0,699), high (0,700-0,799) and very high (0,800–1) [16].

The IVS is an index built to complement the IDHm and to identify overlaps of social exclusion and vulnerability indicative situations in a given territory. It is composed by three dimensions that represent state provisions assets whose deprivation negatively impacts on population welfare conditions and that are measured by a sixteen indicators set. The three dimensions are: urban infrastructure (measured by indicators related to water and sewage supply, to garbage collection and to travel time from home to labor), human capital (measured by indicators related to child mortality rate, to young population school flow, to adult population schooling and to young mothers proportion) and income and labor (measured by indicators related to the per capita household income, to unemployment, to informal occupation, to financial dependence on the elderly and to people from 10 to 14 years activity). Thus, the IVS aims to be an indicative of goods and services provision failures by the Brazilian State. It is available for all geographic levels: country, regions, states and municipalities. The range of social vulnerability measured by the index is: very low (0–0,200), low (0,201-0,300), medium (0,301-0,400), high (0,401-0,500) and very high (0,501–1) [17].

The names of municipalities were validated and those that could not be safely related to an existing municipality were excluded from the study.

To assess the (de)concentration of health and, therefore, the equity degree achieved by the set of lawsuits in health in Minas Gerais, descriptive and statistical analysis were conducted.

To identify the general profile of all municipalities that had residents who benefited from lawsuits in health in Minas Gerais, central tendency measures (mean and median) were used for the description of quantitative variables as well as the standard deviation, the minimum and maximum and the percentiles 25 and 75. Relative and absolute frequency were used for the description of the following adopted categorical variables: number of beneficiaries (1–2/above 3), municipality’s populational size (below median/above median), IVS (less vulnerable = below percentile 75/more vulnerable = above percentile 75) and IDHm (high-very high/low-medium).

To verify what factors were associated with a larger number of beneficiaries of lawsuits in health in a municipality, a binary logistic regression was conducted between the dependent categorical variable (number of beneficiaries) and the independent ones. Odds Ratios (OR) with the corresponding 95% Confidence Interval (CI) were used to show the strength of associations, and variables with *P*-values of < 0.05 were considered statistically significant. The analysis was made by the software SPSS Statistics Base Screenshot 22.0.

IVS 2010 and IDHm 2010 maps were collected from IPEA’s Social Vulnerability Atlas website and a map marking the main municipalities benefited from the judicialization of health care in Minas Gerais, from 1999 to 2009, was built with TabWin software.

## Results

Out of the 853 Minas Gerais’ municipalities, 399 were registered as residence of at least one of the 6.906 lawsuits beneficiaries in the state from 1999 to 2009. These 399 municipalities concentrated 82,90% of Minas Gerais’ population in 2010. The proportion of lack of information about the beneficiary’s municipality of residence within lawsuits was of 11,5%.

The descriptive analysis of the dependent and independent variables revealed the general profile of the 399 municipalities. The minimum number of identified beneficiaries living in a municipality was 1 (one) while the maximum was 1920. The smallest population size was 1210 inhabitants and the largest one was 2,375,151. The IVS fluctuated from very high to very low and the IDHm varied from low to very high. While the mean number of inhabitants was 40,719.92, 50% of the municipalities had a population up to 14,661 people. Absolute and relative frequencies calculated for dependent and independent categorical variables indicated that 51.9% of the 399 municipalities had 1 or 2 residents that benefited from lawsuits in health, 298 of them showed IVS below 0.323 and 44.4% of them exhibited IDHm high or very high. Table 1 provides detailed information about the descriptive analysis.

**Table 1:** Descriptive analysis of characteristics of the 399 municipalities that had at least 1 beneficiary from lawsuits in health in Minas Gerais from 1999 to 2009

Variables	n	%
*Number of beneficiaries 1999–2009*		
1–2	207	51.9
> 3	192	48.1
Mean (SD)	15.25 (102.78)	–
Median	2	–
Min – Max	1–1,920	–
Percentile 25	1	–
Percentile 75	6	–
*Resident population 2010*		
0–14,661	200	50.1
> 14,661	199	49.9
Mean (SD)	40,719.42 (134,843.954)	–
Median	14,661	–
Min – Max	1,210–2,375,151	–
Percentile 25	7,173	–
Percentile 75	31,883	–
*IVS 2010*		
0–0.322	298	74.7
> 0.323	101	25.3
Mean (SD)	0.2863 (0.0785)	–
Median	0.271	–
Min – Max	0.158–0.56	–
Percentile 25	0.229	–
Percentile 75	0.324	–
*IDHm 2010*		
high – very high	177	44.4
low – medium	222	55.6
Mean (SD)	0.69024 (0.0785)	–
Median	0.693	–
Min – Max	0.536–0.813	–
Percentile 25	0.661	–
Percentile 75	0.723	–

Source: GPES/UFMG’s Judicialization of Health Care 1999–2009 Database; IPEA’s Social Vulnerability Atlas 2010; prepared by the authors

The binary logistic regression revealed that high and very high IDHm (OR = 3045; IC = 1773-5228), IVS below 0.323 (OR = 2044; IC = 1099- 3800) and populational size above 14.661 inhabitants (OR = 6162; IC = 3733-10,171) are statistically associated to a greater number of beneficiaries of lawsuits in health within a municipality. Table 2 displays the findings of the statistical analysis.

**Table 2:** Statistical analysis of socioeconomic factors associated to a number of beneficiaries of lawsuits in health above 3 in a municipality

Variable	Categorization	β	OR	IC 95%	*p*-value
IDHm	High-very high	1.113	3.045	1.773–5.228	< 0.001
	Medium-low		1		
IVS	0–0.322	0.715	2.044	1.099–3.800	0.024
	> 0.323		1		
Resident population	> 14.661 inh.	1.818	6.162	3.733–10.171	< 0.001
	0–14.661 inh.		1		

Source: GPES/UFMG’s Judicialization of Health Care 1999–2009 Database; IPEA’s Social Vulnerability Atlas 2010; prepared by the authors

**Fig. 1:**
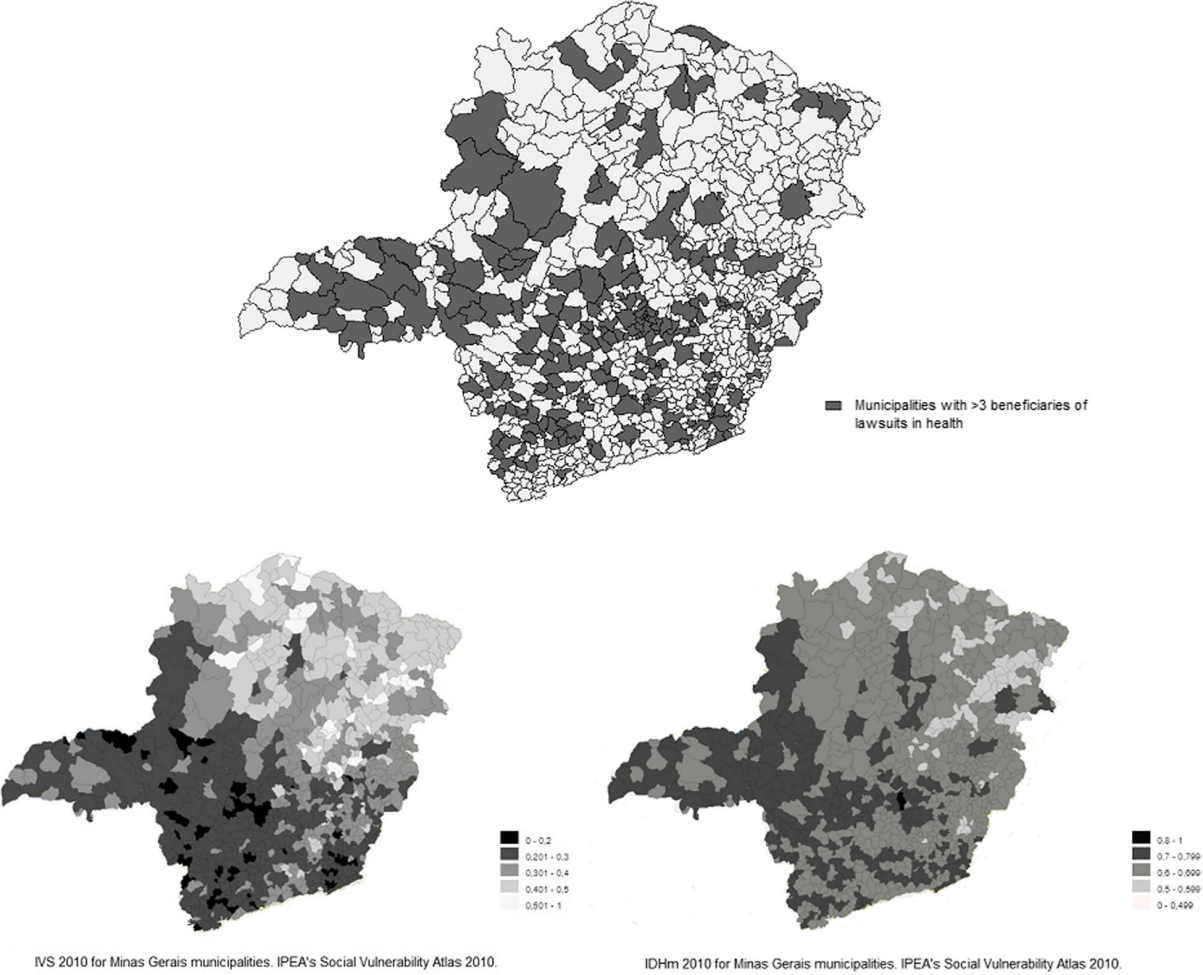
Maps of Minas Gerais regarding the 192 municipalities with number of beneficiaries of lawsuits in health over than 2, the IVS 2010 and the IDHm 2010

Maps of Minas Gerais concerning the IVS 2010 and the IDHm 2010 were compared to a map of the state where the 192 municipalities with number of beneficiaries of the judicialization of healthcare over than 2 are marked (Fig. 1).

## Discussion

In societies marked by inequities, as the Brazilian one, health protection necessarily passes through its social determinants discussion, once there is convincing evidence of association between a population’s diseases distribution and its socioeconomic conditions [4, 18, 19]. Therefore, according to Duarte [14], in the impossibility of redistributing diseases among populations, health actions that are proposed to be equitable must seek to attenuate factors that contribute to health inequities.

Vieira-da-Silva and Almeida Filho [18] point that the State can formulate policies that are promoters of more or less equity. So, the Judiciary, as part of the State, when proposing itself as an auxiliary force for the fulfilment of the constitutional right to health, must also be alert to the health outcomes achieved by its set of decisions.

In this study, complex socioeconomic evaluation indexes and descriptive and statistical analysis were adopted to substantiate the investigation about the judicialization of healthcare effects over equity. It was observed, then, that most citizens benefited by the phenomenon from 1999 to 2009, in Minas Gerais, lived in municipalities that registered better socioeconomic conditions. A statistically significative association was observed between larger number of beneficiaries of a municipality and a high or very high municipal human development, a larger municipal populational size and a lower municipal social vulnerability. It suggests, therefore, that the set of judicial decisions in health, opposed to the principle of equity, had favored a concentration of health resources in these municipalities for the first ten years of experience with the judicialization of healthcare phenomenon in Minas Gerais.

Furthermore, the comparison of the IVS 2010 map, the IDHm 2010 map and the map with marked municipalities with number of beneficiaries above 3 suggests that, in the studied period, the judicial performance in Minas Gerais could not reach and benefit citizens living in municipalities where interventions of the State were most needed.

These outcomes are alike the data presented by Ferraz [20] in a study published in 2011 which points out that there was a concentration of lawsuits in the richest cities and states of Brazil – 93,3% of the litigation was located within the 8 states with the highest IDH (above 0,8). Ferraz [20] suggests this result can be explained by the inequity of access to courts and good lawyers. The author reflects that, for example, for every individual lawsuit demanding access to a medicine, there may be a great number of unrepresented non-litigant interested parties. Thus, limited health resources have been reallocated in favor of few privileged individuals even if their needs are not considered public health priorities [20].

Brinks and Forbath [21] reflect that the Brazilian State has always favored privileged groups and hasn’t addressed structural issues to overcome historical inequalities. Therefore, it is not a surprise to figure out that the judicial intervention has also failed to benefit the unprivileged Brazilians.

The distributive justice notion, usually associated with equity, prescribes that primary social goods, as opportunities and wealth, should be equally distributed among society. Once verified the market failure in distributing social wealth in an egalitarian way, the State would intervene to correct this mistake. In order to ensure equity, the State could even adopt a positive discrimination in favor of disadvantaged groups [14, 18, 22, 23]. Thus, from the results found in this study, it arises a hypothesis of a contrary positive discrimination tendency - in favor of advantaged groups - within the scope of the judicialization of health care.

As well as the distributive justice notion assigns the State the attribution of correcting market failures [14, 18, 22, 23], the justification for judicial intervention in the political field lies in an argument of public policies failures necessity of correction [24, 25]. So, since the IVS index aims to signal state failures to provide essential goods and services for the Brazilian population well-being, comparing the IVS and the judicialization of healthcare maps raises also a questioning about the adequacy of judicial performance in health for the corrective function proposed by it.

When thinking about equity and distributive justice, another point has to be discussed from this study’s results. As meeting the judicial demands against SUS requires public resources from a common budget for financing all health actions and services offered by the Brazilian public health system [15], the concentration of lawsuits beneficiaries in municipalities with better socioeconomic conditions doubly suggests damage to equity: the judicial performance set would not only be benefiting advantaged groups but would also be potentially harming disadvantaged groups by determining reallocation of health resources in order to comply with court orders.

Once SUS’ organization is decentralized and all government spheres are responsible for ensuring the right to health [26], states and municipalities consist in gateways for the judicialization of health care that are closer to the population, what makes it difficult to identify a national level overview of the phenomenon. Being municipalities the federated entity with lower income, the financial impact of the judicialization of health care may be more significant: in 2013, while the budget to purchase basic medicines for the entire population of Tubarão - state of Paraná - was about US$ 279,288, the municipality spent US$ 280,467 on the attendance of health judicial sentences [22].

Duarte [14] indicates that, among the factors that determine the equity degree within a health system, the way of distributing financial resources is one of the most important. Therefore, this impact of the judicialization of health care on health resources distribution must be deeply investigated, once, according to Achoki and Lesego [27], health financing changes have intended and unintended consequences that can negatively affect health outcomes when they are not holistically appreciated.

However, the configuration of the judicialization of healthcare phenomenon which has been consolidated in Brazil, through individual demands for access to health technologies - especially medicines [8, 11, 12, 20, 21, 28], makes it difficult for the Judiciary to evaluate collective results of its decisions. This conformation of the judicialization of healthcare also meets Fortes’ [24] say that in late capitalism societies, citizens’ individual yearnings tend to overlap collective interests, what hinders an effective implementation of equity principle. Thus, we wonder about the possibility of achieving an equitable judicial performance in health when it is based on individual demands.

According to Brinks and Forbath [21, 28], different courts’ interventions forms have different effects on politics. There are lawsuits challenging political issues of structural nature, on the contrary of individual demands, but Flood and Gross [9] point that courts are more conservative in intervening in them, despite being quite open to individual demands in some countries, like Brazil.

In this country, for example, the Judiciary has been provoked to manifest about the constitutionality of the Constitutional Amendment 95/2016 (EC 95/16) that froze public expenditure for 20 years, including in health - what has been considered highly harmful to SUS by specialists [29, 30]. Without facing improper financial restrictions due to EC 95/16, the judicialization of health care will fight for resources of an already reduced budget, increasing probabilities of damage to equity by a judicial performance centered on individual demands.

Other countries, however, have experienced other conformation of the judicialization of health care phenomenon [21, 28, 31]. The Colombian Judiciary, for example, after having extensively experienced individual demands and conflicted with the executive branch [28], started addressing what Garavito [31] called “structural demands” and could figure out the process of fulfilment of economic and social rights in a broader way. Having seriously considered the budgetary issues, the Colombian Judiciary invited interested parties to discuss the health system funding, what resulted in a completely and more equitable restructure of the public health system [28, 31]. From the perspective of structural cases, it is possible that equity issues in health become more evident and that judicial intervention become more assertive and capable of helping to ensure access to health goods and services without distributive distortions.

According to Brinks and Forbath [21], the activity of litigating social and economic rights is relatively new and we are only starting to understand its real effects. There may be some indirect positive political consequences of litigation – even individual ones [21, 28] - that are difficult to assess. However, looking at the Brazilian experience in comparison with other countries as Colombia, and recognizing that an equitable assurance of the right to health passes through its social determinants coping [3, 4, 6, 24], we consider it more reasonable to think (and to suggest) that the judicialization of healthcare, once presented as a corrective tool for health public policies failures, should be driven to structural issues of collective effects that hold up the maintenance of diseases and social goods uneven distribution among society - for example issues regarding health systems financing and intellectual property of litigated technologies. When anchored in its observed conformation from 1999 to 2009 in the state of Minas Gerais, Brazil, the judicialization of healthcare, as partly demonstrated in this study, does not seem to be an auxiliary activity for the fulfilment of an equitable right to health.

Lack of records about all beneficiaries’ municipality of residence and about beneficiaries’ individual socioeconomic conditions account for limitations of this study. However, we consider the investigation methodology suitable to substantiate the developed discussion.

## Conclusions

The study points that the judicialization of healthcare in Minas Gerais, Brazil, from 1999 to 2009 did not reach municipalities where State intervention necessity was more evident. On the contrary, the phenomenon favored a concentration of health resources in places with better socioeconomic profiles.

Quality records about all beneficiaries’ municipality of residence and their individual socioeconomic conditions are important for more conclusive analysis. However, despite study limitations, we believe the results to be sufficient indication that the judicialization of healthcare in Minas Gerais, from 1999 to 2009, was not an auxiliary tool for the fulfilment of an equitable right to health. New longer-term studies – including qualitative ones - must be conducted to assess not only the direct but also the indirect effects of the judicialization of healthcare on the distribution of health resources in Brazil and other countries.

From the findings of this investigation, we question the judicial performance suitability for its proposed corrective function as its possibility to assist in the assurance of an equitable right to health from individual demands. However, looking at the experience of other countries as Colombia, we ponder that when states fail to ensure equitable public policies, structural litigation may be an opportunity for the Judiciary to help addressing issues that affect the distribution of social goods and public services among society.

The Brazilian Judiciary has been provoked to intervene in structural issues that limit SUS’ capacity to fulfill a comprehensive, universal and equitable right to health. The judicial questioning of the constitutionality of the EC 95/16 is one of the main examples in this sense. Nonetheless, the Constitutional Court does not seem ready – or willing – to start addressing core issues that prevent Brazil from managing its marked social inequalities.

## Abbreviations

EC 95/16: Constitutional Amendment 95/2015
SUS: Brazilian National Health Service
